# The association between Parkinson’s disease and melanoma: a systematic review and meta-analysis

**DOI:** 10.1186/s40035-015-0044-y

**Published:** 2015-11-03

**Authors:** Pei Huang, Xiao-Dong Yang, Sheng-Di Chen, Qin Xiao

**Affiliations:** Department of Neurology & Institute of Neurology, Ruijin Hospital affiliated to Shanghai Jiao Tong University School of Medicine, Shanghai, 200025 China

**Keywords:** Parkinson’s disease, Melanoma, Meta-analysis, Odds ratios

## Abstract

**Objective:**

To assess the association between Parkinson’s disease (PD) and melanoma via systematic review and meta-analysis.

**Methods:**

Comprehensive search in PubMed, Web of Science, Embase and four China databases (SinoMed, WanFang data, CNKI and VIP database) of epidemiologic evidences on PD and melanoma published before April 30, 2015. Studies which reported risk estimates of melanoma among PD patients or risk estimates of PD in patients with melanoma were included. Pooled odds ratios (ORs) with 95 % confidence intervals (CIs) were calculated by random-effects models. Heterogeneity across studies was assessed using Cochran Q and I^2^ statistics. Subgroup analyses and sensitivity analyses were conducted to evaluate sources of heterogeneity. Subgroup analyses were done according to temporal relationship, geographic region and gender respectively. We assessed publication bias using the Begg and Egger test. In addition, study appraisal was done using a scale for observational studies to ensure the quality of evidence.

**Results:**

We identified 24 eligible studies on PD and melanoma with a total number of 292,275 PD patients: the pooled OR was 1.83 (95 % CI 1.46–2.30) overall, subgroup analyses by temporal relationship showed that risk of melanoma after PD diagnosis was significantly higher (OR 2.43, 95 % CI 1.77–3.32), but not before the diagnosis of PD (OR 1.09, 95 % CI 0.78–1.54). Subgroup analysis by geographic region showed that increased risk of melanoma in PD was found both in Europe (OR 1.44, 95 % CI 1.22–1.70) and in North America (OR 2.64, 95 % CI 1.63–4.28). Gender-specific subgroup analyses did not show difference between men (OR 1.64, 95 % CI 1.27–2.13) and women (OR 1.38, 95 % CI 1.04–1.82) in the risk of melanoma. In addition, we found the risk of non-melanoma skin cancers in PD was slightly higher (OR 1.20, 95 % CI 1.11–1.29) than general population. It was impossible to evaluate the association between PD and melanoma according to use of levodopa or gene polymorphism via meta-analysis since few observational or cohort studies have focused on it.

**Conclusions:**

An association between PD and melanoma was confirmed. Most of the evidences were of high quality, and the conclusion was robust. Further research is needed to explore the mechanisms underlying this relationship.

**Electronic supplementary material:**

The online version of this article (doi:10.1186/s40035-015-0044-y) contains supplementary material, which is available to authorized users.

## Introduction

A growing number of evidences suggest that people with Parkinson’s disease (PD) have a decreased risk of almost all cancers [1–3]. However, the incidence of melanoma is strikingly higher in patients with PD than that in general population [4–6]. Some case reports have linked the use of levodopa (L-dopa) with the occurrence of malignant melanoma [7, 8], while some reviews reported that there was apparently no effect of L-dopa on the risk for malignant melanoma [9, 10]. The underlying mechanism that link PD with melanoma is not clear, but it has aroused lots of interests.

The epidemiology of melanoma focus on well-known risk factors, such as skin color, hair color, gender and eye pigmentation, moreover, ultraviolet (UV) exposure is also a risk factor for melanoma [11]. White population is considered to have higher prevalence of melanoma than the dark population and the prevalence is higher in men than in women [11, 12]. Considering the varieties of the reported studies in study design, patient characteristics and geographical region, the discrepancy in the results may be related to insufficient statistical power of individual studies. Here we conducted a systematic review and meta-analysis to evaluate the relationship between PD and melanoma. We also evaluated the non-melanoma skin cancer risk ratio among patients with PD. Subgroup analyses with regard to temporal relationship between PD and melanoma diagnosis, geographic region and gender were also done.

## Methods

### Search strategy

We conducted a systematica search of the published original studies that reported the association between skin cancers and PD. We searched PubMed, Web of Science, Embase and four China databases (SinoMed, WanFang data, CNKI and VIP database) for literature published before April 30, 2015. We combined medical subject heading (MeSH) terms including “PD, Melanoma, Skin Neoplasms”, “Carcinoma, Basal Cell” “Carcinoma, Squamous Cell” and “Keratosis, Actinic” and text terms including “Parkinson disease, Parkinsons disease, Parkinson’s disease, melanoma*, skin neoplasm*, basal cell carcinoma, squamous cell carcinoma, actinic keratosis and skin cancer*” as search strategy. Restrictions were made to observational epidemiologic studies involving humans.

### Eligibility criteria

Studies were included if they reported an estimate of association between PD and melanoma, e.g. relative risk (RR), odds ratio (OR), standardized incidence ratio/event ratio (SIR/SER) with 95 % confidence intervals (CIs) or data sufficient to calculate them. Then studies in the form of case report, review, meta-analysis and meeting abstract were excluded. Next we evaluated the potential studies by screening titles and abstracts and the reference lists of all the articles to find other relevant articles and to further exclude unqualified studies. In details, two of the coauthors PH and XDY independently evaluated the study titles and abstracts, and disagreements in the study selection were discussed among the coauthors until consensus was reached. Also duplicate counting of events was carefully avoided.

### Data extraction and classification

Two of the coauthors PH and XDY independently searched the literature and extracted the relevant information [see Additional file 1] including: name of first author, study data, study design, sample size, patient characteristics, geographical region, adjusted risk estimates with corresponding 95 % CIs and other relevant study characteristics. Attempts were made to contact authors to obtain gender-specific ORs and 95 % CIs if they were not provided in the original publications. Disagreements were resolved by consensus.

Subgroup analyses were performed according to temporal relationship between PD and melanoma diagnosis (study design), geographical region and gender. We also evaluated the relationship between non-melanoma skin cancers and PD.

### Assessment of study quality

Two of the coauthors PH and XDY independently scored each study [see Additional file 2] according to the following five criteria items [13]: 1) definition of PD diagnosis (clearly defined using generally accepted criteria; less well-defined with partial use of generally accepted criteria; other or no criteria applied; with scores of 2, 1, 0, respectively); 2) validation of PD diagnosis (confirmed by neurological exam; confirmed by chart review; no attempt to validate; with scores of 2, 1, 0, respectively); 3) adjustment for confounding factors (multiple factors including age and smoking; age alone or along with factors other than smoking; none; with scores of 2, 1, 0, respectively); 4) source and definition of cancer diagnoses (based on chart or pathology review; based on ICD code/death certificate or derived from population register; none of the above; with scores of 2, 1, 0, respectively); and 5) representativeness of cases (includes the great majority of cases in a defined population or a representative population-based sample; includes only some cases in a population leading to an under-representative sample, with scores of 1, 0, respectively). Potential total scores ranged from 0 to 9. Studies scored 0–3, 4–6 and 7–9 were of low, medium and high quality. Final consistency of scores was achieved through consensus.

### Statistical analysis

The STATA, version 12.0 (StataCorp, College Station, TX) was used to perform the analyses. We made no distinction between varying measures of association (SIR/SER, OR or RR) reported in different studies and used OR for all the studies. The pooled ORs with corresponding 95 % CIs were calculated with Der Simonian and Laird random-effects model [14]. Heterogeneity was assessed using Cochran’s Q and I^2^ statistics, the percentages of I^2^ around 25, 50 and 75 % mean low, medium and high heterogeneity, respectively [15]. Subgroup analyses and sensitivity analyses were conducted to evaluate sources of heterogeneity. Subgroup analyses were done according to temporal relationship, gender and geographic region, respectively. Publication bias was evaluated using Begg rank correlation test and the Egger regression asymmetry test [16, 17].

## Results

### Search results and study characteristics

**Fig. 1:**
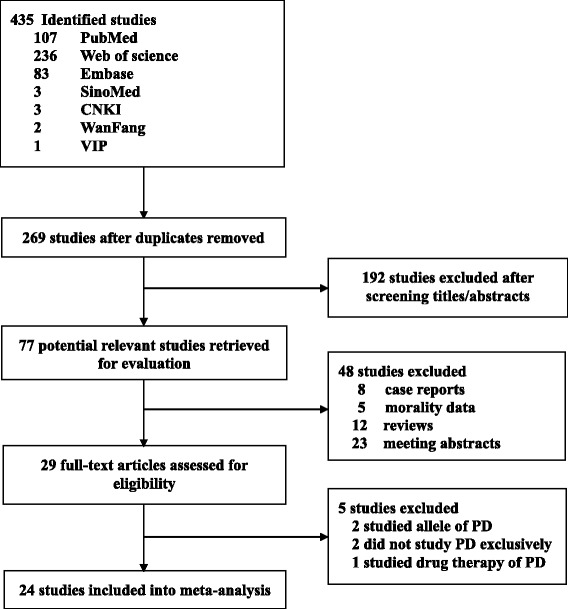
Flow diagram: Risk estimates on the association between PD and melanoma

Twenty-four articles fulfilled our inclusion criteria and were included in our meta-analysis with a total number of 292,275 PD patients (Fig. 1). Among the 24 studies there were 16 cohort studies [1–6, 10, 18–26], six case control studies [27–32] and two cross-section studies [33, 34]. Nine of the studies also provided information on non-melanoma skin cancers and PD [3, 5, 10, 18, 19, 23, 27–29], and one study [33] only provided the risk of skin cancers in PD patients. Most of the studies were conducted in Europe and North America, only two studies were conducted in Asia [4, 26], and one study in Oceania [31].

### Association between melanoma and PD according to temporal relationship

**Fig. 2:**
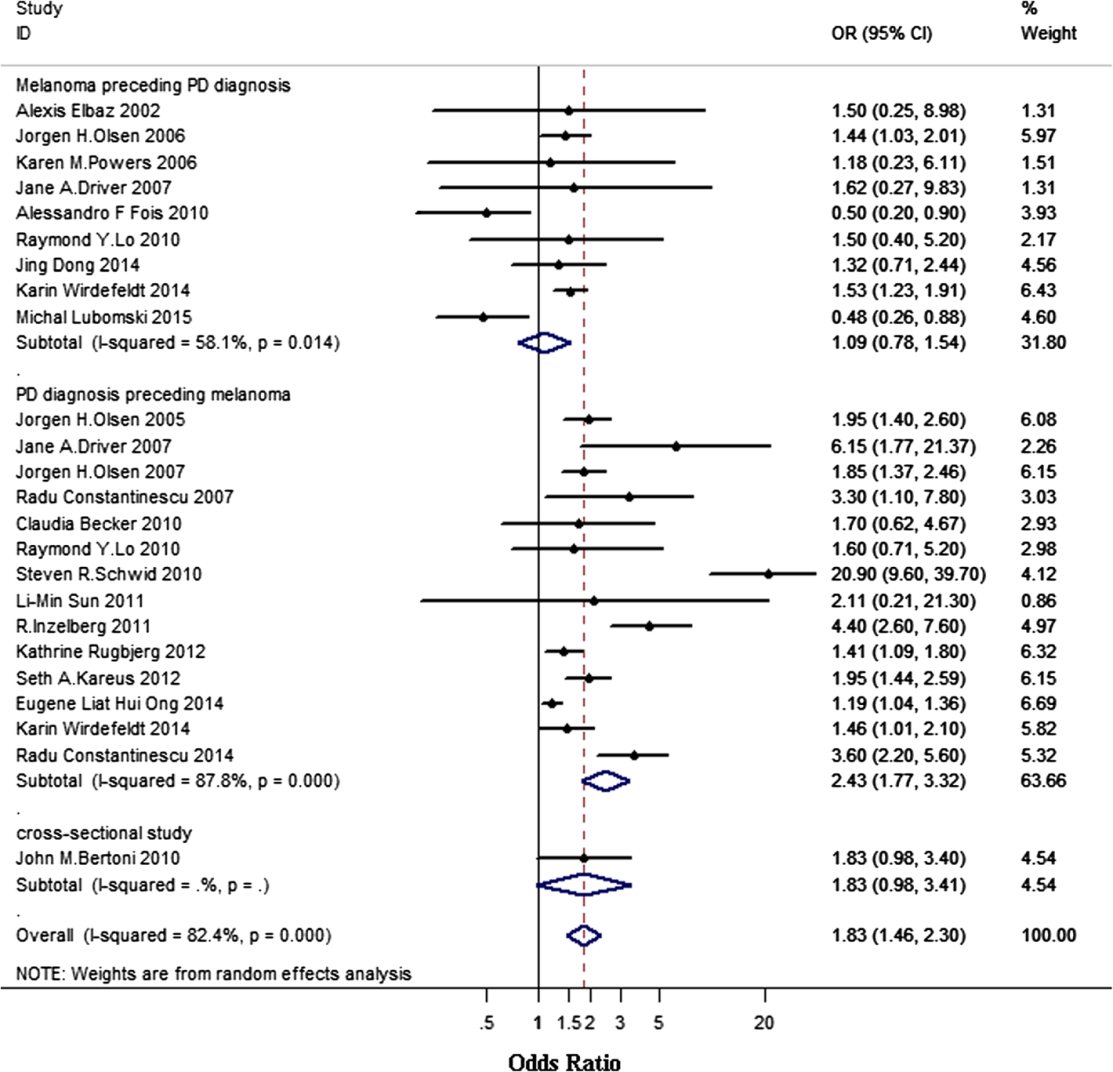
Association between melanoma and PD according to temporal relationship. Subtotal = pooled odds ratios (ORs) within each subcategory. Overall = pooled OR for all studies. Squares indicate study specific ORs; error bars indicate 95 % confidence intervals (CIs); diamonds indicate ORs and 95 % CIs from pooled analyses

A total of 22 studies were included in the analysis, we divided all the 22 studies into three subgroups according to temporal relationship (study design), including “PD diagnosis preceding melanoma (cohort studies)” with 14 studies [1–6, 10, 19–22, 24–26], “melanoma preceding PD diagnosis (case–control studies)” with nine studies [3, 23, 24, 27–32] and “co-occurrence of PD and melanoma (cross-sectional studies)” with only one study [34]. Two studies provided data separately for melanoma before and after PD diagnosis [3, 24]. As shown in Fig. 2, the overall pooled OR was 1.83 (95 % CI 1.46–2.30), however, there was a significant heterogeneity across studies (I^2^ = 82.4 %, PQ < 0.001). Subgroup analysis showed that the pooled OR for “melanoma preceding PD diagnosis” group was 1.09 (95 % CI 0.78–1.54), with evidence of moderate heterogeneity (I^2^ = 58.1 %, PQ = 0.014). People with PD had increased risks (OR 2.43, 95 % CI 1.77–3.22) of melanoma compared with those without PD as shown in the “PD diagnosis preceding melanoma” group, with a significant heterogeneity across studies (I^2^ = 87.8 %, PQ < 0.001). Only one study reported the co-occurrence of PD and melanoma and the OR was 1.83 (95 % CI 0.98–3.40). We examined the source of heterogeneity by excluding the study of poor quality [23]. After excluding this study from the analysis, the pooled OR appeared significantly stronger (OR 1.93, 95 % CI 1.54–2.42), but the heterogeneity was still high (I^2^ = 82.0 %, PQ < 0.001). When we further exclude the study that reported the highest OR (OR 20.90) [25] and the study that reported the lowest OR (OR 0.48) [31], the pooled OR was 1.80 (95 % CI 1.52–2.14), and the heterogeneity was reduced by nearly 22 % (I^2^ = 64.5 %, PQ < 0.001). In detail, the heterogeneity in “melanoma preceding PD diagnosis” group was completely eliminated (I^2^ < 0.1 %, PQ = 0.999), while the heterogeneity in “PD diagnosis preceding melanoma” group was significant (I^2^ = 78.4 %, PQ < 0.001). To further examine the source of heterogeneity across studies in “PD diagnosis preceding melanoma” group, we did subgroup analysis by geographic region (see the following).

### Geographic region-specific analysis

**Fig. 3:**
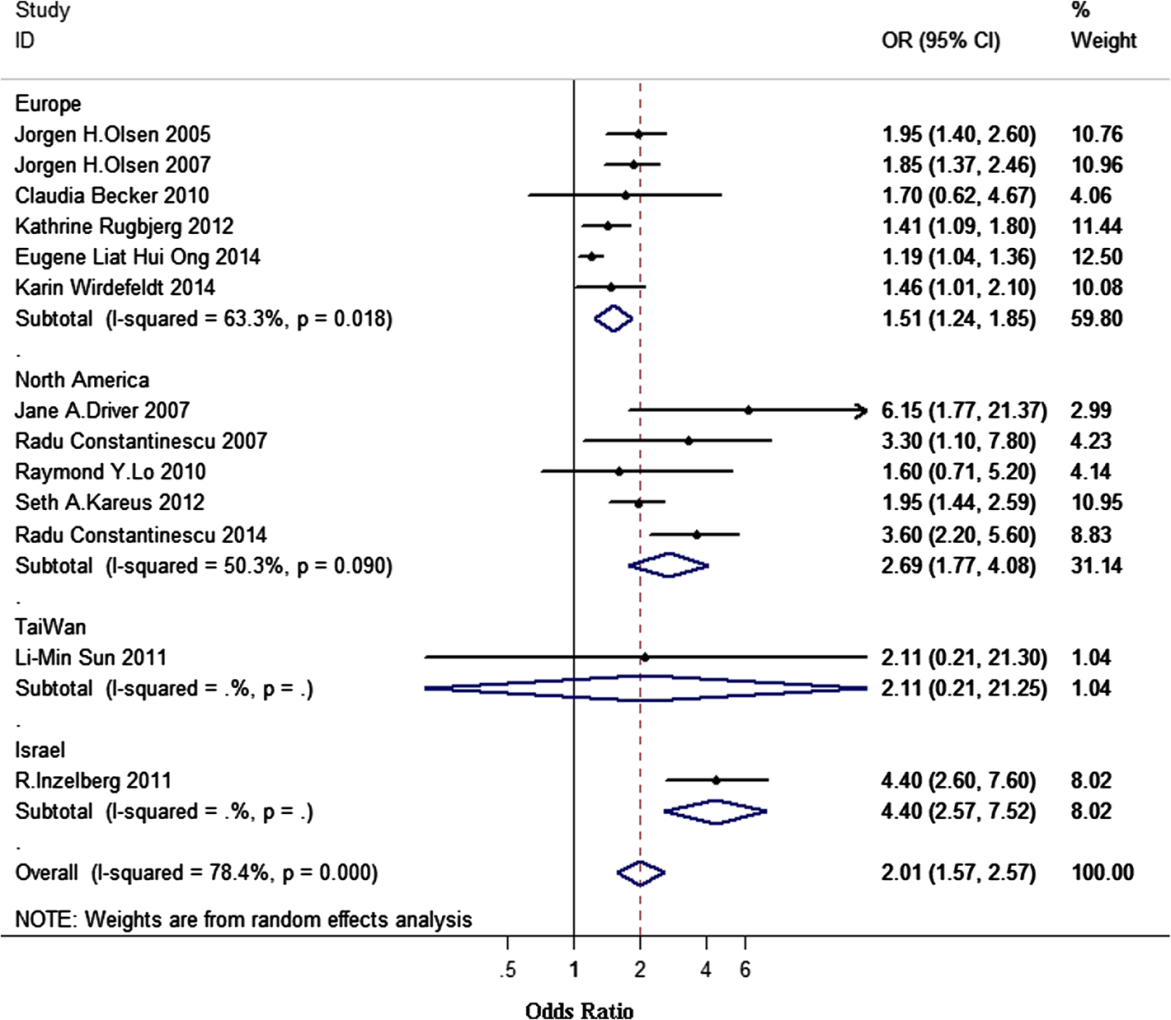
Association between melanoma and PD according to geographic region. Subtotal = pooled odds ratios (ORs) within each subcategory. Overall = pooled OR for all studies. Squares indicate study specific ORs; error bars indicate 95 % confidence intervals (CIs); diamonds indicate ORs and 95 % CIs from pooled analyses

In the subgroup analysis by geographic region (Fig. 3), the overall pooled OR were 2.01 (95 % CI 1.57–2.57), with high heterogeneity (I^2^ = 78.4 %, PQ < 0.001). Most of studies were done in Europe and North America, the pooled OR for Europe was 1.51 (95 % CI 1.24–1.85) with medium heterogeneity (I^2^ = 63.3 %, PQ = 0.018) and 2.69 for North America (95 % CI 1.77–4.08) with relatively lower heterogeneity (I^2^ = 50.3 %, PQ = 0.09). Both Israel (OR 4.40, 95 % CI 2.57–7.52) and Taiwan (OR 2.11, 95 % CI 0.21–21.25) only had one study.

### Gender-specific analysis

**Fig. 4:**
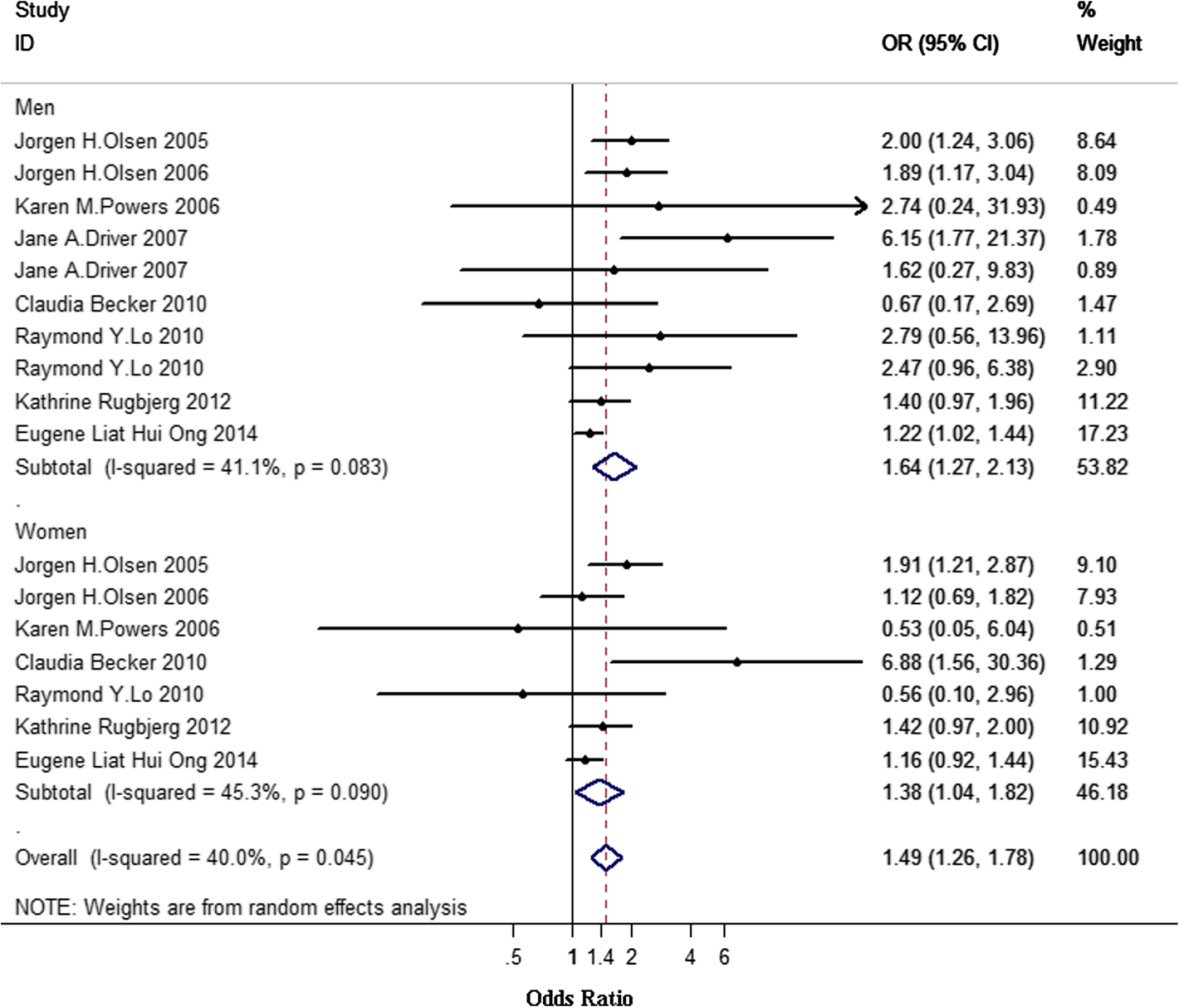
Association between melanoma and PD according to gender. Subtotal = pooled odds ratios (ORs) within each subcategory. Overall = pooled OR for all studies. Squares indicate study specific ORs; error bars indicate 95 % confidence intervals (CIs); diamonds indicate ORs and 95 % CIs from pooled analyses

In the subgroup analysis by gender, we included 9 studies for men [2, 5, 19, 21, 22, 24, 28–30] and 7 studies for women [2, 5, 19, 22, 24, 28, 29]. In men, one study provided data separately for melanoma before and after PD diagnosis [24]. Our results (Fig. 4) showed that the association between melanoma and PD was similar in both men (OR 1.64, 95 % CI 1.27–2.13) and women (OR 1.38, 95 % CI 1.04–1.82), with evidence of moderate heterogeneity (I^2^ = 40.0 %, PQ = 0.045).

### Non-melanoma skin cancers and PD

**Fig. 5:**
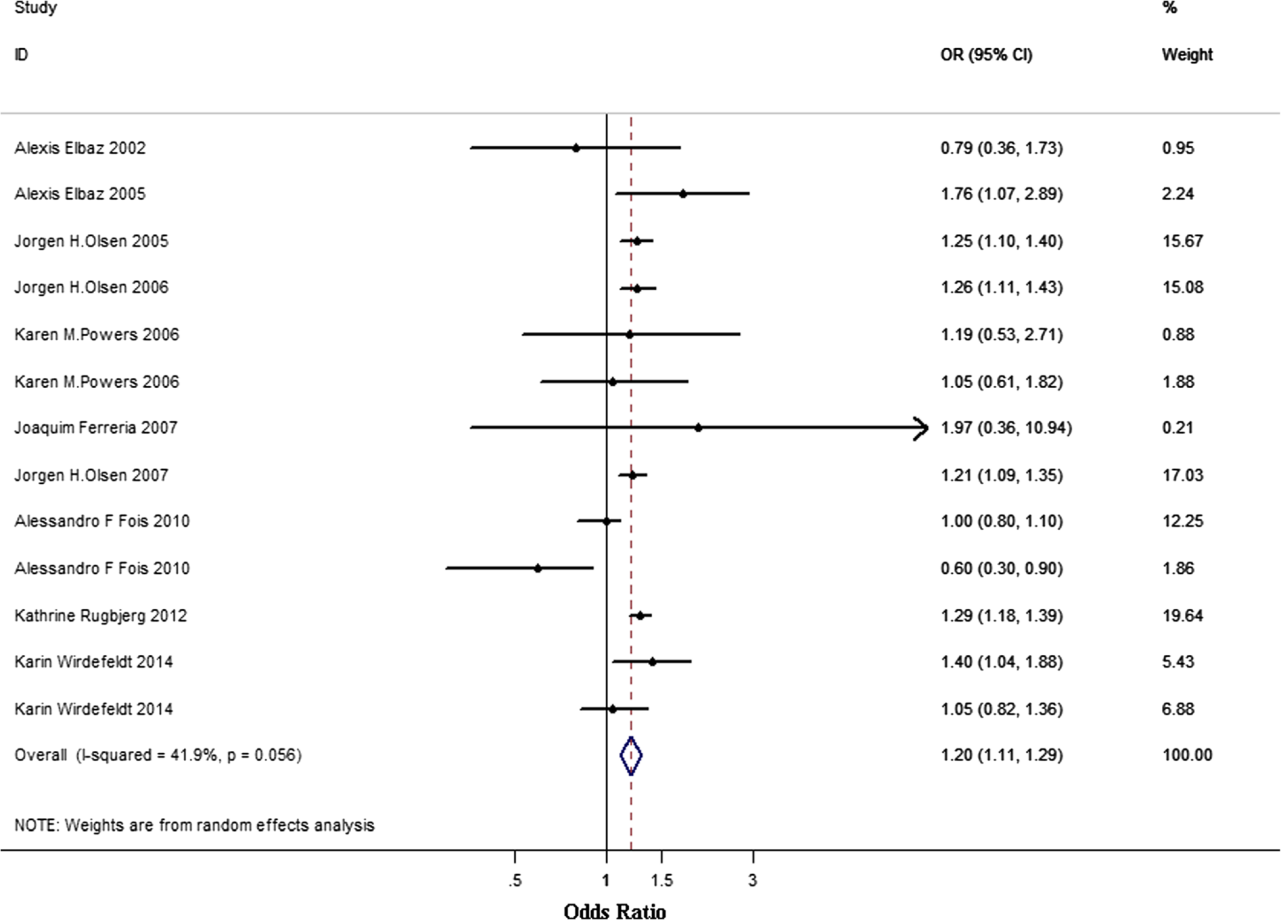
Association between non-melanoma skin cancers and PD. Subtotal = pooled odds ratios (ORs) within each subcategory. Overall = pooled OR for all studies. Squares indicate study specific ORs; error bars indicate 95 % confidence intervals (CIs); diamonds indicate ORs and 95 % CIs from pooled analyses

We included 10 studies [3, 5, 10, 18, 19, 23, 27–29, 33] that provided information on non-melanoma skin cancers and PD (Fig. 5). Two studies provided the ORs both before and after PD [3, 23]. One study provided only gender-specific ORs [29]. The cumulative estimated risk associated with non-melanoma skin cancers was 1.20 (95 % CI 1.11–1.29) with evidence of moderate heterogeneity (I^2^ = 41.9 %, PQ = 0.056). After excluding the study of low quality [23], the overall pooled OR was 1.25 (95 % CI 1.19–1.32) and the heterogeneity was completely eliminated (I^2^ < 0.1 %, PQ = 0.713).

### Evaluation for publication bias

The publication bias was detected from results of Begg test and showed no significant evidence for bias (*P* = 0.503). The Egger test also did not show significant publication bias (*P* = 0.125).

## Discussion

Parkinson’s disease (PD) is a neurodegenerative disorder, characterized by depletion of dopamine in the striatum and loss of melanin-positive, dopaminergic neurons in the substantia nigra pars compacta (SNpc) [35, 36]. On the other hand, cancer is a large group of diseases, characterized by uncontrolled growth of cells and the ability of metastasis. Although PD and cancer seem to drive the cells to different outcomes, that is either degeneration or overproliferation, the association between PD and cancer has been supported by plenty of epidemiologic studies, which have shown that the incidences of most cancers are lower in PD patients compared with controls [1–3]. However, the incidence of melanoma is strikingly higher in patients with PD than that in general population [4–6]. The underlying mechanism that link PD with cancer is not clear, but it has aroused lots of interests.

The results of our meta-analysis show a definite association between PD and melanoma. Since studies included were of different study design, the heterogeneity across studies was high. Subgroup analysis by study design reduced some part of heterogeneity and the heterogeneity further decreased after subgroup analysis by geographic region. The result that risk of melanoma in PD patients is significantly higher than that in controls is in agreement with the result of previous meta-analysis [37]. We involved more eligible evidences and carefully assessed the quality of evidences which made the results much more confirmed and we also added comparison between different regions in the risk of melanoma. Possible mechanisms of the close association between PD and melanoma are as follows.

Melanin is the primary determinant of skin and hair color in human and more melanin pigment means darker in skin color. Melanin exists in neurons within SN is termed as “neuromelanin”; it is a protective factor that can save neurons from oxidative stress. Abnormalities in melanin can cause various skin cancers, including melanoma, while PD is correlated with abnormalities in neuromelanin, it suggests that PD closely links to melanoma through melanin [38]. Previous studies show melanin can reduce the susceptibility of skin to develop into melanoma [39, 40]. However, decreased level of neuromelanin in SN is associated with increased susceptibility of the neurons to oxidative stress and impaired motor functions [41, 42]. Thus, melanin acts as a key connection between PD and melanoma.

As the synthesis of dopamine and melanin share biomedical pathways and some case reports have found PD patients developed local melanoma after initiation of L-dopa therapy [7, 43-45], it is plausible that some scholars link the high prevalence of melanoma in PD to L-dopa therapy [46]. However, findings from recent clinical and epidemiologic studies suggest that the link between L-dopa therapy and melanoma was coincidental rather than causal [10, 20]; it is PD that increases the risk of melanoma rather than L-dopa therapy. However, to get reliable results we need to do meta-analysis, but it was impossible yet since there were only two observational studies evaluating the association between use of L-dopa and the incidences of melanoma in PD [10, 20]. Opponents argue that melanocytes have not been shown to be able to incorporate external L-dopa. In addition, it has been shown that dopamine is toxic for melanocytes in vitro [47]. Furthermore, the increased rate of melanoma before the diagnosis of PD weakens the hypothesis that melanoma may be induced by the treatment of PD [28]. On the other hand, we found that PD may also increase the risk of non-melanoma skin cancers [18, 19, 28]. The association between PD and non-melanoma skin cancers also supports the conclusion of L-dopa as a causal factor, unless the biochemical pathway including L-dopa is common to all types of skin cancer. Other anti-Parkinsonian drugs, such as selegiline and CEP-1347, are also found to have no influence on the association between PD and melanoma [20, 25].

Another potential explanation is that PD and melanoma have genetic correlation. PD is characterized by genetic susceptibility to environmental toxins due to decreased enzymatic detoxification [48, 49]. Lack of expression of enzymes encoded by GSTM1 can lead to death of neurons related to PD [50-52]. In addition, CYP2D6 polymorphism and VDR polymorphism also play important roles in the pathogenesis of PD [51, 53, 54]. Considering that high frequency of polymorphisms of CYP2D6 or the polymorphism of VDR, and null for GSTM1 gene in melanoma patients [55-57], it is plausible that changes in GSTM1, CYP2D6 and VDR genes may increase the risk for both PD and melanoma. Furthermore, some PD-related genes, such as Parkin [58, 59], alpha-synuclein [60, 61], LRRK2 [62, 63] and DJ-1 [64, 65], were also found to play critical roles in the pathogenesis of melanoma. To sum up, mutations of those genes can increase the risk of both PD and melanoma. However, scholars have found recently that the LRRK2 G2019S mutation carriers had statistically significant increased risks for non-skin cancers and there was no association with melanoma [66]. Thus, to get reliable results we need more studies focusing on the association between gene polymorphism and the risk of melanoma in PD to allow for meta-analysis.

In addition, autophagy deficits have been linked to neurodegenerative disease, such as PD, causing problems in the clearance of injured mitochondria and aggregated proteins [67, 68], whereas autophagy is also suppressed in melanoma [69, 70]. Defective autophagy can promote melanoma initiation and progression by causing impaired antigen presentation and melanoma immune escape. Thus, reduction of autophagy plays an important role both in PD and in melanoma. Whether PD links to melanoma through defective autophagy still needs much more direct evidences.

As to the risk factors of the development of melanoma, previous meta-analysis have shown family history of melanoma, high density of freckles, light skin color and fair hair color as major risk factors for melanoma [71]. White population is considered to have higher prevalence of melanoma than the dark population. However, since studies are mainly from Europe and North America, it is impossible to evaluate the correlation between race and the risk of developing melanoma in PD. More observational studies focusing on the association between PD and melanoma from other race are needed. In addition, with regard to geographic region, increased risk of melanoma in PD was found both in Europe (1.51 times) and in North America (2.69 times). The frequency of melanoma seems to be lower in Europe than in North America, it may be due to the following reasons. In Europe, melanoma incidence varies among north/south and east/west regions with Northern Europe gaining the most profoundly increase in melanoma incidence. Variations in Europe could be explained by differences in skin phenotype as well as in sun-exposure behaviors. Also it may be in part due to differences in case reporting and registration. Only half of European countries have good quality cancer registries, leading to the possibility of melanoma being under-reported in certain countries.

We also performed gender-specific meta-analysis since incidence of melanoma had been shown to be lower in women than men [12]. However, our results show that the connection between PD and melanoma is similar in both men and women, that is, increased risk of melanoma in PD was found both in males and females.

We found that PD patients had slightly increased risk of non-melanoma skin cancers than general population (OR 1.20, 95 % CI 1.11–1.29). After excluding the study of low quality [23], the heterogeneity was completely eliminated and no significant change was found in OR (1.25, 95 % CI 1.19–1.32). However, the results of a previous meta-analysis [37] indicated that there was no association between PD and non-melanoma skin cancers (OR 1.11, 95 % CI 0.94–1.30). Our results only indicate a possible association between non-melanoma skin cancers and PD. These conflicting results must be resolved by including more reliable studies into meta-analysis in the future. In addition, we found OR for non-melanoma in PD was only 1.2; there was no significant increase. One of the common causative factor for melanoma and non-melanoma skin cancers is long-term sun exposure, it may be hypothesized that PD patients are more sensitive to sun-exposure-induced skin lesions. The increased frequency of non-melanoma in PD may be due to a disease-specific susceptibility. However, melanoma occurs at a higher frequency in PD patients than non-melanoma skin cancers, it seemed that besides sun-exposure as a common risk factor, the shared biochemical pathways between the synthesis of both dopamine and melanin made PD patients more likely to develop melanoma. In the future, it is necessary to see the difference in the risk factors between PD patients who developed melanoma and those who developed non-melanoma skin cancers.

### Limitations

There were several limitations in our meta-analysis. Firstly, most of the studies included were not focusing on the association between PD and melanoma; melanoma was mostly evaluated along with other cancers. Moreover, the number of cases with both PD and melanoma is usually small. Lastly, the majority of these studies did not collect enough data on risk factors for us to explore potential explanations.

## Conclusion

In conclusion, melanoma occurs more frequently among patients with PD. Most of the evidences were of high quality, and the conclusion was robust. According to the references, most scholars consider that there are no strong evidences supporting the idea that dopaminergic therapy shall increase the risk of melanoma in PD. However, reliable conclusion will need more studies focusing on the association between L-dopa use and the development of melanoma in PD. The positive association between PD and melanoma may be explained by pigmentation changes in melanin and/or melanin synthesis enzyme, genetic correlations or autophagy deficits.

## Additional files

Additional file 1:
**Characteristics of studies included in the Meta-analysis.** Including information of first author, year of publication, country, study design, number of patients and controls, duration of follow-up. (PDF 74 kb) Additional file 2:
**Quality assessment of studies included in the Meta-analysis.** Criterion include five parts: 1) definition of PD diagnosis; 2) validation of PD diagnosis; 3) adjustment for confounding factors; 4) source and definition of cancer diagnoses; 5) representativeness of cases. Potential total scores ranged from 0 to 9. (PDF 127 kb)
