# Supplementary material for: The association between Parkinson’s disease and melanoma: a systematic review and meta-analysis
Source: Transl Neurodegener. 2015 Nov 3;4:21. doi: 10.1186/s40035-015-0044-y (PMC4631109; doi:10.1186/s40035-015-0044-y)
Supplement: Additional file 1: — Characteristics of studies included in the Meta-analysis. Including information of first author, year of publication, country, study design, number of patients and controls, duration of follow-up. (PDF 74 kb) [file 40035_2015_44_MOESM1_ESM.pdf]

Characteristics of studies included in the Meta-analysis

First author and publication year	Country	Study design	No. of total PD patients	No. of PD patients with melanoma	No. of PD patients with non-melanoma skin cancer	No. of total control	No. of control with melanoma	No. of control with non-melanoma skin cancer	Duration of follow-up (years)
Alexis Elbaz 2002	UK	case-control	196	3	13	196	2	16	NA
Alexis Elbaz 2005	UK	cohort	196	NA	39	185	NA	27	NA
Jorgen H.Olsen 2005	Denmark	cohort	14088	44	292	NA	NA	NA	NA
Jorgen H.Olsen 2006	Denmark	cohort	8090	46	343	32320	128	1100	NA
Karen M.Powers 2006	USA	case-control	352	3	NA	484	3	NA	NA
Jane A.Driver 2007a	USA	case-control	487	NA	NA	487	NA	NA	NA
Jane A.Driver 2007b	USA	cohort	487	9	NA	487	0	NA	Cases:5.2 Controls: 5.9
Joaquim Ferreria 2007	Portugal	cross-sectional	150	0	4	146	0	2	NA
Jorgen H. Olsen 2007	Denmark	cohort	14088	48	328	NA	NA	NA	25
Radu Constantinescu 2007	USA	cohort	800	5	NA	NA	NA	NA	6
Alessandro F Fois 2010	Southern England	cohort	4355	9	185	NA	NA	NA	3.2-3.4
Claudia Becker,	Switzerland	cohort	188	9	NA	278	6	NA	11

2010									
John M.Bertoni 2010	North America	cross-sectional	2106	92	1	NA	NA	NA	5
Raymond Y.Lo 2010	USA	cohort	692	6	NA	761	5	NA	4.3
Steven R.Schwid 2010	USA	cohort	806	10	NA	NA	NA	NA	NA
Li-Min Sun 2011	Taiwan	cohort	4957	NA	NA	19828	NA	NA	3
R.Inzelberg 2011	Israel	cohort	1395	20	11	NA	NA	NA	5
Kathrine Rugbjerg 2012	Denmark	cohort	20343	65	586	NA	NA	NA	5.7
Seth A.Kareus 2012	USA	cohort	2998	48	NA	NA	NA	NA	NA
Eugene Liat Hui Ong 2014	UK	cohort	219194	227	NA	NA	NA	NA	NA
Jing Dong 2014	USA	cohort	808	18	NA	1623	26	NA	NA
Karin Wirdefeldt 2014	Sweden	cohort	11786	177	172	58930	586	725	51
Radu Constantinescu 2014	USA	cohort	1737	19	NA	NA	NA	NA	6
Michal Lubomski 2015	Australia	cohort	5637	14	NA	8143	42	NA	NA
