# Supplementary material for: The association between Parkinson’s disease and melanoma: a systematic review and meta-analysis
Source: Transl Neurodegener. 2015 Nov 3;4:21. doi: 10.1186/s40035-015-0044-y (PMC4631109; doi:10.1186/s40035-015-0044-y)
Supplement: Additional file 2: — Quality assessment of studies included in the Meta-analysis. Criterion include five parts: 1) definition of PD diagnosis; 2) validation of PD diagnosis; 3) adjustment for confounding factors; 4) source and definition of cancer diagnoses; 5) representativeness of cases. Potential total scores ranged from 0 to 9. (PDF 127 kb) [file 40035_2015_44_MOESM2_ESM.pdf]

Quality assessment of studies included in the Meta-analysis

First Author and published year	Definition of PD diagnosis	Validation of PD diagnosis	Adjustment	Source and definition of cancer diagnoses	Representative ness of cases	Total score
Alexis Elbaz 2002	2	2	2	1	0	7
Alexis Elbaz 2005	2	2	2	2	0	8
Jorgen H.Olsen 2005	1	1	1	1	0	4
Jorgen H.Olsen 2006	1	1	1	1	1	5
Karen M.Powers 2006	2	2	2	0	0	6
Jane A.Driver 2007a	2	2	1	1	1	8
Jane A.Driver 2007b	1	2	2	2	1	8
Joaquim Ferreria 2007	2	2	1	2	1	8
Jorgen H.Olsen 2007	1	1	2	1	0	5
Radu Constantinescu 2007	1	1	1	1	0	4
Alessandro F Fois 2010	0	1	1	1	0	3
Claudia Becker 2010	2	2	1	1	1	7
John M.Bertoni 2010	1	2	2	2	0	7
Raymond Y.Lo 2010	1	1	2	1	1	6
Steven R.Schwid 2010	2	2	0	2	0	6
Li-Min Sun 2011	1	1	1	1	1	5
R.Inzelberg 2011	2	2	1	2	0	7
Kathrine Rugbjerg 2012	1	1	2	1	0	5
Seth A.Kareus 2012	1	1	1	1	0	4
Eugene Liat Hui Ong 2014	1	1	1	1	0	4

Jing Dong 2014	1	2	1	1	1	6
Karin Wirdefeldt 2014	1	1	1	1	1	5
Radu Constantinescu 2014	2	2	1	1	0	6
Michal Lubomski 2015	1	1	1	0	1	4
